# Development of a Novel Tailless X-Type Flapping-Wing Micro Air Vehicle with Independent Electric Drive

**DOI:** 10.3390/biomimetics9110671

**Published:** 2024-11-03

**Authors:** Yixin Zhang, Song Zeng, Shenghua Zhu, Shaoping Wang, Xingjian Wang, Yinan Miao, Le Jia, Xinyu Yang, Mengqi Yang

**Affiliations:** 1 School of Automation Science and Electrical Engineering, Beihang University, Beijing 100191, China; zhang_yixin@buaa.edu.cn (Y.Z.); song_zeng@buaa.edu.cn (S.Z.); 18308335530@163.com (S.Z.); shaopingwang@buaa.edu.cn (S.W.); 22376239@buaa.edu.cn (L.J.); yang_xinyu@buaa.edu.cn (X.Y.); yangmengqi@buaa.edu.cn (M.Y.); 2 Ningbo Institute of Technology, Beihang University, Ningbo 315800, China; 3 Tianmushan Laboratory, Hangzhou 310023, China; 4 School of Mechanical Engineering, Tsinghua University, Beijing 100084, China

**Keywords:** bio-inspired robot, flapping-wing air vehicle, tailless control, lattice Boltzmann method, clap-and-peel

## Abstract

A novel tailless X-type flapping-wing micro air vehicle with two pairs of independent drive wings is designed and fabricated in this paper. Due to the complexity and unsteady of the flapping wing mechanism, the geometric and kinematic parameters of flapping wings significantly influence the aerodynamic characteristics of the bio-inspired flying robot. The wings of the vehicle are vector-controlled independently on both sides, enhancing the maneuverability and robustness of the system. Unique flight control strategy enables the aircraft to have multiple flight modes such as fast forward flight, sharp turn and hovering. The aerodynamics of the prototype is analyzed via the lattice Boltzmann method of computational fluid dynamics. The chordwise flexible deformation of the wing is implemented via designing a segmented rigid model. The clap-and-peel mechanism to improve the aerodynamic lift is revealed, and two air jets in one cycle are shown. Moreover, the dynamics experiment for the novel vehicle is implemented to investigate the kinematic parameters that affect the generation of thrust and maneuver moment via a 6-axis load cell. Optimized parameters of the flapping wing motion and structure are obtained to improve flight dynamics. Finally, the prototype realizes controllable take-off and flight from the ground.

## 1. Introduction

Flying animals, despite being tiny in size compared to most air vehicles, shows superior flying skills with their flapping wings [1]. It is demonstrated that flapping wings can produce higher lift under certain conditions than conventional aerofoils [2]. Biological observations and fluid analyses [3,4] have led to aerodynamic mechanisms, including rotational circulation [5], leading edge vortex attachment [6] and wake capture [7]. These mechanisms significantly increase the lift during flapping wing compared to the mechanism of lift generation under steady flow. Therefore, the development of bio-inspired flapping-wing micro air vehicle (FMAV) might show potential due to the advantage of their small sizes and high lifts.

Tailless FMAV has the advantage of agility when maneuvering, but they are unstable and require active stabilization mechanisms. Some researches have successfully developed insect-inspired or bird-inspired tailless hoverable robots. Nano Hummingbird [8] developed by AeroVironment, exactly demonstrated the ability to perform the controlled hovering flight, which generates the moments by three servos that modulate the wing twist, similar to the changes of angle of attack in insects. Harvard RoboBee [9], an insect-sized two-wing MAV, achieved controlled flight. Kar’asek et al. [10] developed a hovering flapping-wing MAV with a control mechanism based on flapping amplitude and offset modulation. The prototype can produce pitch moments between -0.7 Nmm and 1.1 Nmm while wings flap at frequencies around 17 Hz and produce a lift of at least 90 mN. A novel tailless flapping wing robot with four pairs of wings is proposed, named Quad-thopter, which implemented a flight time of 9 min or more [11]. Phan et al. [12,13] developed an insect-like tailless FMAV, KUBeetle with four-bar linkage and pulley-string mechanisms, successfully performing a vertical climb. Three sub-micro servos were integrated to realize the pitch, roll, and yaw controls. Nguyen et al. [14] proposed a tailless FMAV with a wing stroke plane modulation mechanism, namely NUS-Roboticbird, which maneuvers by only using its flapping wings for both propulsion and attitude control. Part of the above-mentioned FMAV is a flapping wing configuration with a pair of wings (two wings), which is closer to real organisms (birds or insects) in bionic morphology. Another part is a flapping wing configuration with two pairs of wings (four wings). This type of configuration is different from biological morphology, but has certain advantages in lift generation mechanism. At the same time, limited by the current Micro Electro Mechanical Systems (MEMS) and micro high-energy density battery technology, some insect-scale FMAVs are powered by towing or thin-film solar cells, and there are cable constraints for controllable flight and short endurance. Although another part of the bird-scale FMAV has achieved controllable flight with flapping of two or four wings, and some have even achieved hovering and high-maneuverability flight, the overall structure is relatively complex, and the manufacturing and lightweight design are difficult. In addition, the mechanical transmission efficiency of the flapping wings is reduced, and the endurance of the prototype needs to be improved. These are all different from the original design intention of this article. We hope to design a controllable bionic aircraft with a certain load-bearing capacity and considerable flight endurance.

The clap-and-fling mechanism was utilized to enhance vertical aerodynamic force in these FMAVs [15]. Researchers from TU Delft [16] and Chiba University [17] implemented multiple high-speed cameras to record the chordal deformation of the vertical wing section of the X-type flapping wing. They reconstructed the velocity field information of the fluid domain at the moment of the flapping through Particle Image Velocimetry (PIV). A mechanism similar to clap-and-fling called clap-and-peel was discovered in nature from the butterfly [4,18]. By deploying two wings flapping in counter phase on each side of the FMAV, the clap-and-peel mechanism can occur on both pairs of wings and produce a greater enhancement on the thrust than a single pair do [19]. The aircraft developed in this research similarly utilizes this phenomenon.

Researchers have been delving into the control of FMAVs and proved that hovering flapping flight is inherently unstable and needs to be stabilized actively [20,21]. Flight control research of DelFly II shows that while FMAV with tail has obvious advantages in stability, the use of control surfaces has an important drawback [22]. Furthermore, flapping-wing flight with two wings (common in natural flight animals) is inherently more unstable than the flapping-wing flight with four wings. In order to develop a controlling system that can produce an optimized flight performance, the characteristics and the influence on flight control of the FMAV are investigated in this research.

This paper aims to develop a novel X-type (four-wings) FMAV with the independent driving mode of the left- and right- wing to form the propulsion and attitude adjustment mechanism. To meet the lightweight requirements and achieve considerable maneuverability, FMAVs should be constructed with light but strong materials and specially designed MEMS [8]. Appropriate choices in fuselage and wing materials are made. An ultra-light control electromechanical system for the drive motor and servo was developed to ensure the lightness and agility of the vehicle. In-depth aerodynamics investigation of the FMAV in hovering via computational fluid dynamics (CFD) method, the wing segmentation flexibility is set to a certain extent to simulate the deformation of the rod-membrane wing in chordwise. The qualitative analysis of the generation and development of vortex under the clap-and-peel mechanism in the flapping process were carried out via the visualization of flow field.

The dynamic characteristics of the prototype manufactured in hovering state are quantitatively obtained through experiments. The relationship between the Pulse-Width Modulation (PWM) motor speed control signal and the output flapping frequency is determined. The aerodynamic forces generated by the four wings and the control torque on each axis under different flapping wing control inputs are precisely and quantitatively measured, and the mathematical mapping relationship between them is obtained accordingly. Finally, the reinforced wing without wing veins is proved in the sense that the clap-and-peel mechanism generated by the membrane wing can further enhance the aerodynamic thrust generated by this type of flapping wing (perpendicular to the stroke plane).

Because of the high maneuverability and multiple flight modes of the novel tailless FMAV, the bionic robot is believed to be able to nimbly pass through narrow spaces. As the size of the whole machine becomes smaller and smaller, it will be applied to complex scenarios such as indoor tracking, emergency rescue and disaster relief.

The remainder of this paper is organized as follows: Section 2 presents the design and matrials of the novel FMAV. Section 3 introduces the CFD simulation for the aerodynamics investigation of the FMAV with the rigid segmented wing model in the chord direction. In Section 4, the dynamic characteristics of the vehicle are verified experimentally. Section 5 presents the specific simulation and experimental results and discusses them. Finally, the conclusions and future prospects are presented.

## 2. Design and Materials of the Flying Robot

### 2.1. Conceptual Design

Different from traditional hovering FMAVs, a tailless structure model with wing vector control was adopted in this study. for flapping-wing air vehicle (FAV), in the case of low speed, especially during hovering, the effectiveness of the control surfaces of the tail diminishes drastically, which is embodied in poor attitude control in the presence of external disturbances [23]. Some tailless flying animals, and insects, for example, maintain their stability actively with their flapping wings during flight and ensure maneuverability [24]. Compared with the aircraft with the empennage, more direct flight maneuvering forces and torques can be generated by flapping the wings, and the response of the flying attitude control will be faster. The damping caused by the rudder effect of the empennage on the flight attitude changes will be removed. Moreover, the aircraft will have high maneuverability, similar to flying creatures.

**Figure 1:**
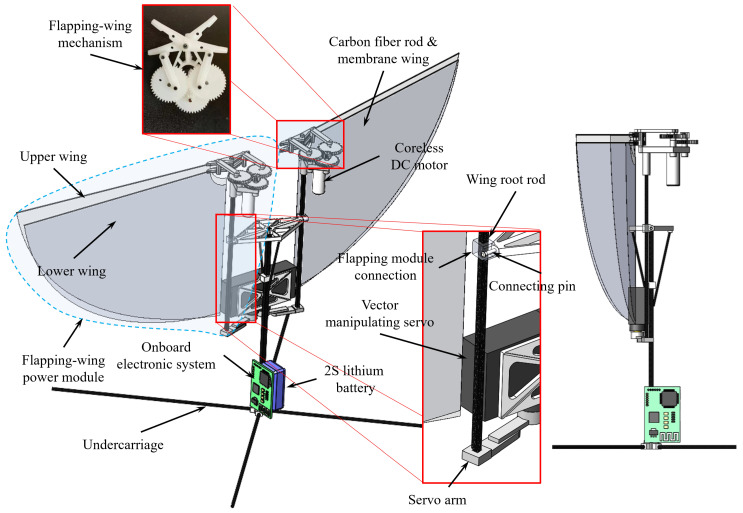
Designed configuration and related details display of the prototype.

As shown in Figure 1, the prototype design and side view include the right wing and its actuation system, the left wing and its actuation system, the main body bracket, the left and right vector manipulating servos, the onboard electronic system, the battery, and the undercarriage. Independent control of the wings on both sides was adopted in this work. The advantage is that it can directly implement pitch and yaw maneuvering for attitude stabilization and control. A pair of ultra-light dual-drive crank-rocker mechanisms are the important components of the prototype, which drive the left- and right-wing, respectively. When flying forward, the balanced state of the wing pair is observed as an X shape in the front view.

The flapping directions of the upper and lower wings are opposite so that the vibration of the wing pair generated during flapping can be suppressed to a certain extent. In this case, the mass of the wings no longer causes unilateral inertial vibration. Since the left and right wings cannot guarantee complete synchronization of the clap-and-peel movement, the peaks of the thrust and flapping moment generated on both sides appear at different times, which will still cause the vibration of the fuselage. The flapping-wing moment is canceled out in the stationary hover, but the thrust generated by a wing pair is not constant in time. Although the thrust generated by a single-sided wing pair is not constant over time, the motor speed is adjusted. The wingbeat frequency is increased so that the average value of the aerodynamic force and moment received by the whole machine is relatively constant, which ensures the stable attitude of the prototype when hovering. The flapping-wing disturbance moment is canceled out.

### 2.2. Hardware and Fabrication

#### 2.2.1. Mechanical Design

The total weight of the aircraft is approximately 26.5 ± 0.1 g, the wingspan (*S*) is about 341 ± 0.1 mm, and the fuselage length is approximately 242 ± 0.1 mm. The machine comprises high-strength carbon fiber rods with round or square cross-sections, 3D-printed photosensitive resin structures, and other components connected by tight fitting and gluing. All structures are fixed on the main carbon fiber rod of the fuselage according to the requirements of the position of the center of gravity (CoG) of the whole machine. From top to bottom are the diamond-shaped fuselage frame, the servo installation support, the battery, and the undercarriage. The structure of the vector propulsion is as follows: In the middle of the prototype, the left and right miniature ultra-light servos (DSP33, POWER-HD, Huizhou, China) weighting 2.9 ± 0.1 g are installed, and the servo shell is directly glued to the installation support. The rudder teeth are connected to the rocker arm, and the other end of the rocker arm is connected to the end of the wing root rod made of carbon fiber. The output rotation of the servo drives the left- and right-wing roots to deflect, which realizes the control of the left- and right-wing root angles ($\xi_{L}$ and $\xi_{R}$) and directly changes the direction of the aerodynamic force on the left- and right-wings. This structure realizes two maneuvers, pitching motion and yaw motion.

The wing root rod is inserted into the flapping-wing mechanism connector, and the connecting hole is tapered. The left and right flapping-wing mechanisms are fixed on the diamond-shaped fuselage frame by connecting pins while retaining the front and back rotation freedom of the wing root rod. The upper end of the wing root rod is connected with the flapping-wing power module. The flapping-wing mechanism rotates around the connecting pin to produce a forward or backward tilt angle, so the stroke plane is changed. The 2.17 g coreless DC motor (6017, XIPHORIX, 6 mm diameter, 17 mm length) decelerates through a two-stage gearbox to amplify the output torque and drive the flapping-wing mechanism to achieve flapping motion. The gear reduction ratio is approximately 25:1. Both the left and right flapping-wing mechanisms include a double crank-rocker structure. Considering the existence of the transmission clearance, the flapping-wing amplitude is determined by the geometric parameters of the mechanical structure to be 81° ± 2°.

The mass distribution of each component of the prototype is shown in Table 1.

#### 2.2.2. Electronic Design

**Figure 2:**
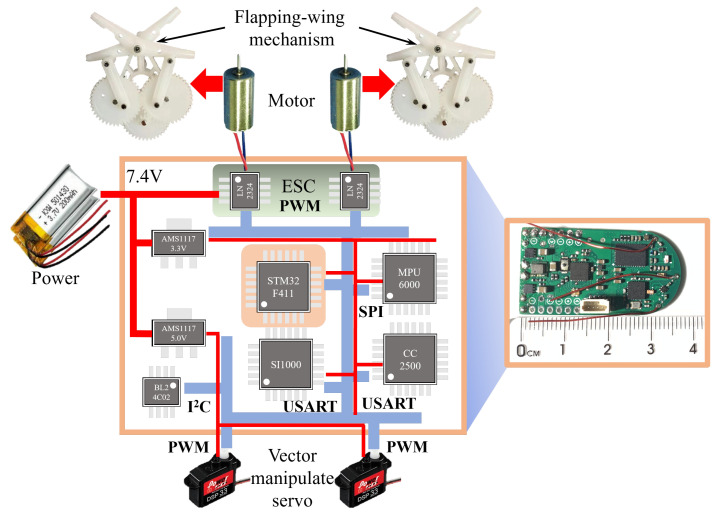
Onboard electronic system structure.

As shown in Figure 2, we have designed a highly integrated onboard electronic system to achieve wireless remote control and attitude feedback stability control. In order to obtain the date of the attitude angle and corresponding angular rates accurately and conveniently, the control board is installed horizontally on the upper surface of the diamond bracket. We built a four-layer control circuit board with an ARM 32-bit Cortex-M4 (STM32F411, STMicroelectronics, Genève, CH) as the core, and reduced the PCB size to (37 mm length, 20 mm width), and the weight was less than 3 g.

For closed-loop feedback, the Inertial Measurement Unit (MPU6000) consists of a 3-axis gyroscope, a 3-axis accelerometer, and a 3-axis digital magnetometer. Two 1S lithium batteries (3.7 V, 200 mAh) are connected in series to output 7.4 V as the power source for flight, which directly outputs to the electronic speed controller (ESC) to drive the motor. 7.4 V is connected to the step-down power regulator chips (AMS1117 3.3 V and AMS1117 5 V) to step down to output 3.3 V and 5 V voltage respectively. 3.3 V is provided to other onboard chips for power supply, while 5 V is used to drive the left and right servos. The total capacity of the battery pack is 200 mAh, which can sustain the flight of the FMAV for more than 3 min.

#### 2.2.3. Wing Design

Previous research revealed that the wings of birds have hard leading edges and that the flexible wings of insects would deform under the action of aerodynamic forces [25,26]. The 1 mm diameter carbon fiber rod with higher stiffness acts as the wing leading-edge drive rod to enhance the spanwise stiffness to ensure the flapping amplitude. The chordwise flexibility of the entire wing should be appropriate: If the wing is too soft to effectively withstand the air force, the aerodynamic thrust will be greatly reduced; if the wing stiffness is too high, the clap-and-peel effect will be weakened, and the aerodynamic thrust generated will also be greatly reduced [19]. The diameter and length of the leading-edge drive rod directly affect the mode shape of the wing under high-frequency flapping motion, which in turn affects the generation of thrust [20].

**Figure 3:**
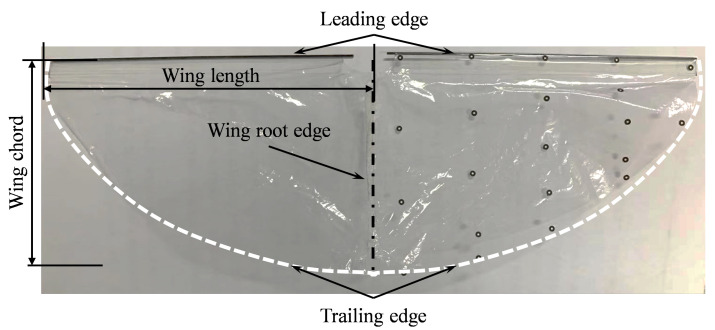
Bionic wings with different membrane materials and wing vein distribution.

Figure 3 shows the design of PVC rod-membrane wing with wing length l=137.5±0.1mm, maximum wing chord c=93.7±0.1mm, thickness h=0.01mm. The black solid line is the leading-edge driven carbon fiber rod, the white dashed line is the wing trailing edge, and the upper and lower wing connecting position is the wing root edge.

#### 2.2.4. Double Crank-Rocker Mechanism

**Figure 4:**
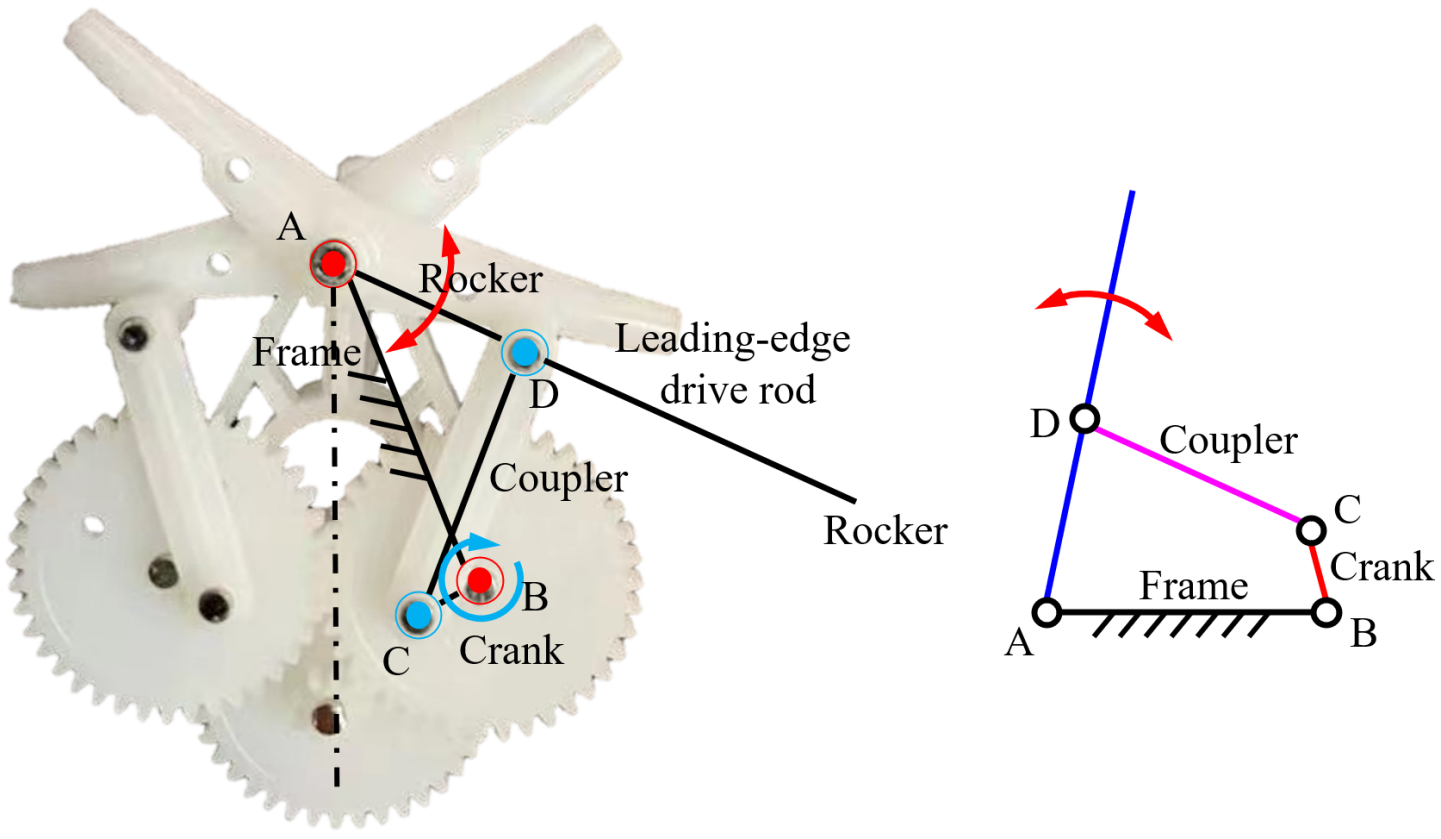
Double crank-rocker mechanism.

Determine the length of the frame as 13.84 ± 0.1 mm, the length of the crank as 2.38 ± 0.1 mm on the last level of the two same diameter gears meshing with each other, the two couplers are hinged to the gear through pins, the length of the coupler is 10.6 ± 0.1 mm, As shown in Figure 4. The other end of the coupler is connected to the rocker of the wing. The length of the rocker is 7.75 ± 0.1 mm. The leading-edge driven rod is inserted into the rocker, and the symmetrical flapping in the opposite direction of the two wings on the same side is realized synchronously.

### 2.3. Clap-and-Peel Mechanism

**Figure 5:**
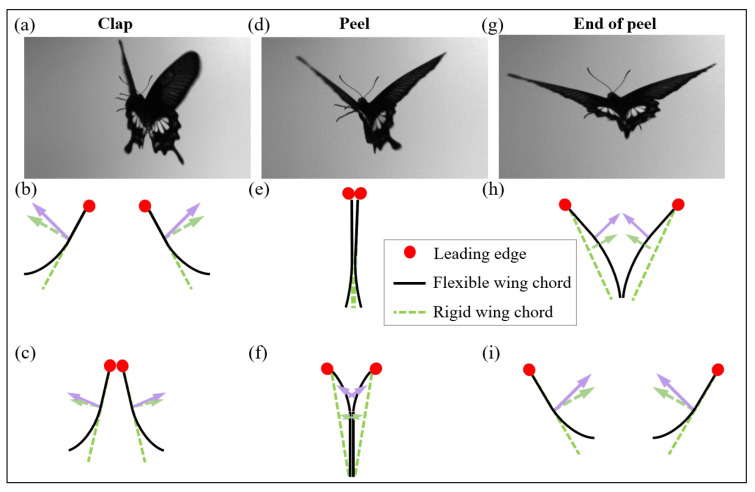
Clap-and-peel mechanism during the flapping wing process. (**a**) Clap of the real butterfly; (**b**) Near clap; (**c**) Leading edges touch together; (**d**) Peel of the real butterfly; (**e**) Completely clap; (**f**) Initial peel; (**g**) End of peel of the real butterfly; (**h**) Trailing edges separate; (**i**) Completely peel.

When the upper and lower wings clap together and open again, due to the chordwise flexibility of the rod-membrane wing, the phenomenon of clap-and-peel has been confirmed to exist [15], similar to the clap-and-fling mechanism proposed by Weis-Fogh [3]. Different flexibility distribution of whole wings in the span and chord direction will cause the trailing edge of the two wings to “stick” when the upper and lower wings open quickly, like a gradual peeling. Figure 5a,d,g are the snapshots of the real butterfly (*Byasa alcinous*) flying forward captured by a high-speed camera from the rear view perspective in three states: clap, peel and end of peel, as marked in the upper middle part of each snapshot. The movement trend of the butterfly’s wings and the deformation of the wing surface are clearly visible.

During flight, when the upper and lower wings clap together, the leading-edge drive rods of the two wings first contact, and then the wings quickly fit together from the leading edge to the trailing edge, and then the two wings separate, and the separation point moves from the leading edge to the trailing edge. The complete clap-and-peel process is shown in Figure 5. The two-dimensional diagram is a simplified graph of the wing profile along the chordwise. The red dot represents the section of the leading edge; the black solid line represents the two-dimensional simplification of the chordwise section of the flexible wing in this paper. Each wing has two sides: the outside surface and the inside surface. The high-speed camera is used to capture the clap-and-peel phenomenon of the X-type prototype in this article. It is divided into 6 typical stages and is represented in Figure 5b,c,e,f,h,i in order. The specific process is as follows: (1) Near clap; (2) Leading edges touch together; (3) Completely clap; (4) Initial peel; (5) Trailing edges separate; (6) Completely peel. This flapping process produces a downward jet of air, and the fuselage gets upward lift due to the interaction force. When peeling off, the wing will also form two large leading-edge vortices, which also provide lift. The green dashed line represents the rigid wing chord of the clap-and-fling mechanism proposed by Weis-Fogh [3]. The purple solid arrow represents the resultant aerodynamic force on the wing during clap-and-peel, while the green dotted arrow represents the resultant aerodynamic force on the wing during clap-and-fling. The magnitude and direction of the resultant aerodynamic force are represented by the length and direction of the arrow. Compared with rigid wings, the clap-and-peel mechanism produced by chordwise flexibility combined with clap and peel motion is an important factor in reducing the drag force generated during the fling and improving lift of this prototype compared to single-wing flapping [27].

### 2.4. Tailless Control Mechanism

The prototype adopts a tailless design, and the flapping wing pairs generate propulsion and have the functions of elevator and rudder. Each wing pair defines a flapping-wing symmetry plane (FWSP), and the carbon fiber wing root is in this plane. The tilt angle of the FWSP relative to the main axis can be changed by driving the wing root deflection through vector maneuvering servos. The magnitude of the thrust produced by the wing pair is related to the wingbeat frequency. The asymmetrical frequency control relies on two independent motors and flapping-wing mechanisms. The essence of the control of the flight state is the comprehensive adjustment of the size and direction of the aerodynamic force generated by the left- and right-wing pairs.

**Figure 6:**
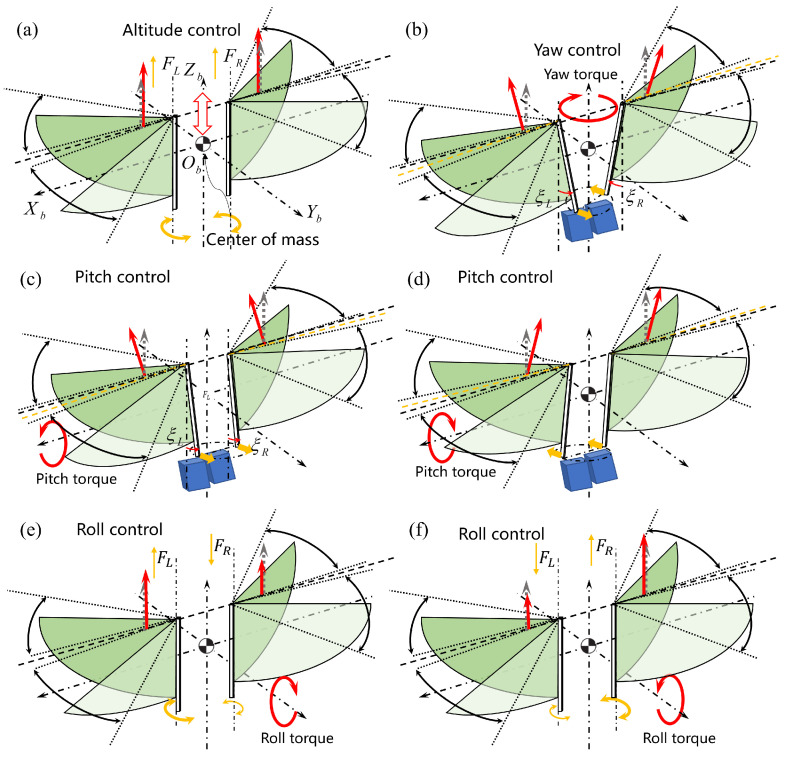
Tailless vector control schematic diagram of the prototype. (**a**) Altitude control; (**b**) Yaw control; (**c**) Pitch control (head up); (**d**) Pitch control (head down); (**e**) Roll control (clockwise); (**f**) Roll control (counterclockwise).

As shown in Figure 6, the dot-dash arrow represents the body-fixed coordinate system, and the origin is defined at the CoG of the robot; the yellow arrow represents the changing trend of the aerodynamic force generated by flapping-wing mechanism, upward means increase and downward means decrease; The red solid straight arrow represents the aerodynamic force vector generated by the left and right wing pairs; the gray dashed straight arrow represents the left and right aerodynamic forces during hovering.

The $\xi_{L}$ and $\xi_{R}$ defined as the angle between the wing root and the main axis of the prototype can be directly changed by the left and right vector maneuvering servo. The root of the membrane wing is constrained by a 1 mm diameter carbon fiber rod. The lower end of the carbon fiber rod is connected to the servo rocker arm. Controlling the servo rocker arm rotation can make the whole wing forward or backward deflection relative to the main axis of the fuselage, thereby generating attitude adjustment moment and achieving attitude control.

Altitude control is achieved by simultaneously changing the aerodynamic forces generated by the left and right flapping-wing power module, that is, synchronous speed regulation of the left and right drive motors, as shown in Figure 6a.

The yaw moment and pitch moment are generated by changing the direction of the aerodynamic force received by the left- and right-wing pairs. Under the condition that the FWSPs of the wing pairs on both sides are parallel to the main axis, the rolling moment is produced by changing the aerodynamic thrust value generated by the left and right wings. The principle of tailless vector control is shown in Figure 6.

1. Yaw control: When the vector maneuvering servos drive the carbon fiber rods on both sides to deviate to the fuselage ventral and dorsal sides, the aerodynamic force generated by the wing pairs projected on the horizontal plane constitutes a pair of force couples relative to the Z-axis, which is the yaw moment. By changing the direction and angle of the rocker arm rotation, the direction, and magnitude of the force couples can be changed to achieve yaw control, as shown in Figure 6b;
2. Pitch control: When the carbon fiber rods on both sides of the wing roots are driven to the same side of the fuselage, front or rear, the left- and right-wing pairs generate the same aerodynamic moment on the X-axis to change the angle of attack of the fuselage. Controlling the magnitude and the sign of $\xi_{L}$ and $\xi_{R}$ can produce different pitching moments. When deflecting to the ventral side, the FMAV raises its head, and when deflecting to the dorsal side, the FMAV lowers its head. The aircraft can maneuver in this way when the hovering mode switches to the forward or backward fast-level flight mode, as shown in Figure 6c,d;
3. Roll control: In the case of determining the geometric parameters, stiffness, and flapping amplitude of the wing, the aerodynamic force can be changed by adjusting the wingbeat frequency. Since the wings on both sides are independently controlled, changing the flapping frequency on one side can change the average thrust of that side. The average thrust on the left and right sides is different, and a rolling moment is generated on the Y-axis. Increasing the difference between the flapping frequencies on both sides to increase the rolling moment can even achieve rapid rolling motion similar to insects, as shown in Figure 6e,f.

Since the flight manipulation of the tailless vehicle is highly coupled, the tilt of the FWSP will inevitably affect the aerodynamic force in the vertical direction. It is necessary to maintain the lift by adjusting the motor speed while controlling the attitude.

### 2.5. Close-Loop Attitude Stabilization and Control

Hovering flapping-wing aircraft is unstable, especially when the tail wing is removed, it needs to actively generate pitch torque ($T_{x}$), roll torque ($T_{y}$), and yaw torque ($T_{z}$) around the main axis of the fuselage to maintain stability [9]. The main goal is to achieve controllable flight of the first prototype through a microelectronic system with extremely limited mass. The onboard microcontroller of the bionic robot in this paper integrates an attitude angle sensing unit, which can be used as feedback information. Therefore, in the selection of the controller, we focus on the design of the flapping-wing aircraft PD controller based on attitude angle feedback in reference [12].

**Figure 7:**
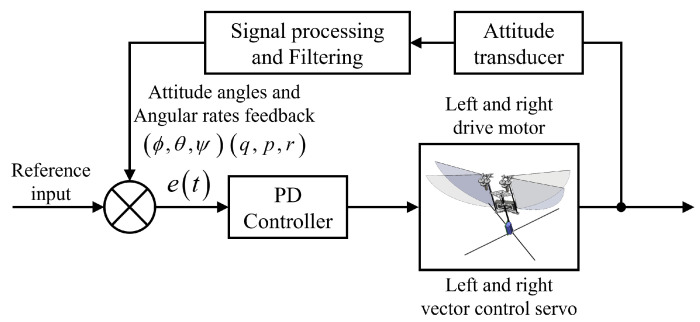
PD attitude feedback control system for the X-type tailless FMAV.

Active feedback control must be considered to achieve stable flight of the tailless FMAV. Due to the unsteady and non-linear characteristics of flapping-wing air vehicle, many control methods have been applied to stability enhancement control. Among them, the NUS-Roboticbird [14], Defly [22], Kubeetle [12] and RoboButterfly-I [28] all adopted a PD controller based on attitude angle feedback and proved to be effective for FAV stable flight. A feedback control system with proportional (P) and derivative (D) terms is designed based on the hardware for sensing attitude angles and angular rates, and is applied to the attitude stability control of the prototype, as shown in Figure 7.

For each of the pitch, roll and yaw motions, their attitude angles (θ, ϕ, ψ) and corresponding angular rates (*p*, *q*, *r*) measured via transducers are designated as excitations *e*(*t*) in the corresponding motion. With these excitations, the commanding signal *u*(*t*) in each direction is produced in the controller according to the formula $u\left(t\right)=k_{p}e\left(t\right)+k_{d}\frac{d}{dt}e\left(t\right)$, where in the coefficients $k_{p}$ and $k_{d}$ are to be tuned in experiments to generate optimized results. Compared with other controllers, this simple PD controller has been successfully verified in many FMAV prototypes that achieve controllable and stable flight.

## 3. CFD Simulation

To reveal the impact of flapping motion, the deformation of wing chordwise on the aerodynamic characteristic of X-type FMAV, the CFD method is used to simulate the vortex structure, further investigate the unsteady aerodynamic lift mechanism. The lattice Boltzmann method (LBM) is used to carry out the detailed fluid simulation analysis, which is proved to be as effective as previous research [29,30].

### 3.1. Computational Setup for CFD Simulation

The kinematics parameters affecting lift and thrust generation and their value rules are obtained, and the optimal parameters are found out under the condition of determining flight speed. The vorticity ω (curl of the fluid velocity field) is used to characterize the three-dimensional (3D) flow field domain [5], and the isosurface is observed to visualize the flow field structure around the simplified FMAV model. Then, the detailed vortex formation and development in the flapping process are analyzed. The 3D structures of the leading-edge vortex (LEV), wing tip vortex (WTV), and trailing edge vortex (TEV) are analyzed via the vortex structure corresponding to the flapping stroke, and the formation and variation laws of vortices during the flight are observed to explain the mechanism of high lift further. The definition of ω is as follows:

(1) $$\omega =\left|\vec{\nabla }\times \vec{V}\right|$$

(2) $$V=\sqrt{V_{x}^{2}+V_{y}^{2}+V_{z}^{2}}$$

where $\vec{\nabla }$ and $\vec{V}$ represent the gradient operator of a vector and the velocity vector of the fluid, respectively. *V* is the velocity value of the fluid. $V_{x}$, $V_{y}$ and $V_{z}$ are the magnitudes of the velocity components of the fluid in the *x*, *y* and *z* directions respectively.

After the kinematics law of the multi-rigid model is imported, the 3D model and the Adams file of motion simulation result are imported into Xflow. In Xflow, the corresponding simulation environment, the calculation domain, fluid parameters, and model and wall boundary conditions are set as shown in Table 2 below.

**Figure 8:**
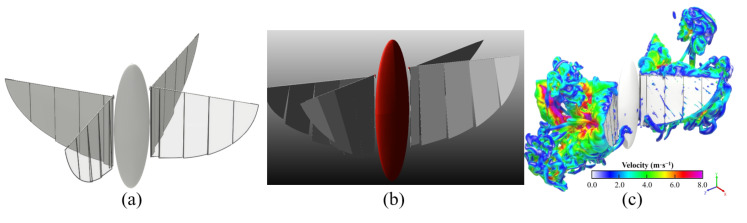
(**a**) Initial location diagram of various parts of prototype model in Xflow; (**b**) Model of X-type FMAV in Adams; (**c**) Visualization of the vortex structure of the flexible model in the Xflow flow field.

As shown in Figure 8, the flow field during the flapping wing stroke of the aircraft is visualized and analyzed through the vorticity isosurface, and the details of the flow field can be observed.

### 3.2. Geometry and Kinematic Model

The 3D FMAV model designed in Solidworks 2019 with complete assembly is imported into Adams 2017. By setting component constraints and joint drive equations, the motion law of the flapping wing is set. The flapping amplitude $A_{f}$, the wingbeat frequency *f*, and the lagging motion law of the segmented rigid body are determined. As shown in Figure 8, the fuselage is simplified into a flat, ellipsoidal rigid body, with two pairs of wings hinged on both sides of the body. The wing length is designed to be 130 mm, and the maximum chord length at the root of the wing is designed to be 95 mm.

In order to reproduce the chordwise flexible deformation more realistically during the flapping wing, the rigid wing is divided into five segments in the spanwise direction to achieve multi-body flexibility. Each segment is connected to the hinge of the drive rod of the wing, so that it can oscillate around the leading edge. The five parts from the wing root to the wing tip are represented as $w_{0}$, $w_{1}$, $w_{2}$, $w_{3}$ and $w_{4}$. Because $w_{0}$ is restricted by the carbon fiber rod at the wing root, there is almost no chordwise deformation in this part, and $\theta_{w_{0}}\left(t\right)\;=\;0$.

The X-type FMAV with independent drive vector control for left and right wings designed in this project has functions of free switching of multiple flight modes and complex mission envelope flight. The most basic task is to achieve fixed-point stable hover, which helps realize aerial reconnaissance and fast maneuvering flight. Through the co-simulation of Solidworks, Adams, and Xflow, we have effectively verified and analyzed the aerodynamic characteristics of the X-type flapping-wing flight. The optimal physical parameters by adjusting *f*, $A_{f}$, and the flexibility of the wing surface were obtained.

The flapping kinematic equation of a single wing is set as $\theta_{f}\left(t\right)$, $A_{f}$ is set to 20°, *f* is set to 20 Hz, and the angle between the balance position of the flapping and the clapping position is 21°. To avoid interference, the initial position of the entire model and the amplitude of each rigid segment are slightly adjusted, as shown in Figure 8. The equivalent motion functions of the segmented flexible wing are set as in Equation (3). The corresponding kinematics laws are loaded on each segment to achieve a film-like flexible deformation effect.

(3) $$\left\{\begin{array}{c} \theta_{f}\left(t\right)=20^{\circ }\cos\left(40\pi t\right)+21^{\circ }\\ \theta_{w_{0}}\left(t\right)=0\\ \theta_{w_{1}}\left(t\right)=-3.5^{\circ }\sin\left(40\pi t\right)-2^{\circ }\\ \theta_{w_{2}}\left(t\right)=-8^{\circ }\sin\left(40\pi t\right)-3^{\circ }\\ \theta_{w_{3}}\left(t\right)=-16^{\circ }\sin\left(40\pi t\right)-5^{\circ }\\ \theta_{w_{4}}\left(t\right)=-24^{\circ }\sin\left(40\pi t\right)-5^{\circ } \end{array}\right.$$

## 4. Experimental Verification

### 4.1. Dynamics Experimental Setup

**Figure 9:**
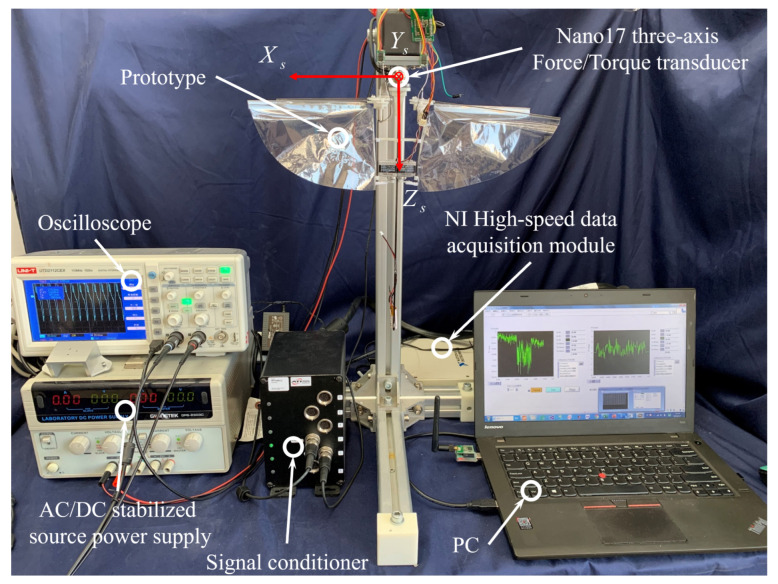
Experiment setup for force, torque and power measurements.

The optimization and iteration experiments of the actual prototype were carried out to improve the maneuverability and aerodynamic efficiency of the FMAV. Figure 9 shows the experimental setup for force, torque, and power consumption measurement.

The experimental system consists of the prototype platform, a prototype control circuit (including ZigBee signal transceiver), and other experimental equipment, as shown in Figure 9, a data acquisition card (DAQ card 6229, National Instruments, Austin, TX, USA) to obtain data, 3-axis force/torque load cell (Nano 17, ATI Industrial Automation, Apex, NC, USA) for the measurement of force and torque, signal conditioning circuit (processing nano17 acquisition data), oscilloscope (UTD2112CEX, UNI-T Instruments, Dongguan, China) for measuring control signals, LED stroboscope (DSS-20, Litong Technology, Suzhou, China) for measuring the actual flapping frequency of the prototype, the DC power supply (which can output a stable voltage of 5–15 V, and can read the real-time current value), a laptop PC reads and records the experimental data. The three-axis force range of Nano 17 transducer (SI-12-0.12): $F_{x}$, $F_{y}$ is 12 N, $F_{z}$ is 17 N, three-axis torque range: $T_{x}$, $T_{y}$ and $T_{z}$ is 120 Nmm, force measurement accuracy is 1/320 N, the torque measurement accuracy is 1/64 Nmm. That is, the resolution of force measurement is 0.318 gf.

Due to the relatively weak rigidity of the first version of the prototype, the high-frequency flapping action will cause the system to vibrate, and the vibration noise signal will interfere with the sensor measurement result, so we use a cut-off frequency of 50 Hz (2 times the flapping frequency). The low-pass FFT filtering method processes the test data. By adjusting the duty ratio of the Pulse-Width Modulation (PWM) wave of the control motor, different swooping frequencies are generated, and the force measurement experiment system is set up to analyze the aerodynamic force and torque generated by the prototype.

The prototype is kept in a vertical attitude. The main carbon fiber rod of the fuselage is connected with the test end of the force transducer through a 3D printing connection structure with bolts. The sensor data is collected through 6 channels of differential signals and NI 6229 is used as the data acquisition device (16 channels of differential or 32 channels of single-ended connection method), which can realize multi-channel high-speed data acquisition.

The sampling frequency is set to 1 KHz, and the data storage frequency is 20 points per second, both can be adjusted according to experimental needs. The DC linear regulator generates a stable 5 V voltage and supplies it to the ESC to drive the coreless motor. In order to ensure the reliability of the data, this paper has carried out multiple experiments, changing the duty ratio of the PWM signal to control the motor speed and the arm angle of the vector servo, the flapping frequency of the left and right wings is measured by a stroboscope. The designed LabVIEW data acquisition host computer records and saves the multi-channel data measured by Nano 17 in real time.

### 4.2. Flight Testing

**Figure 10:**
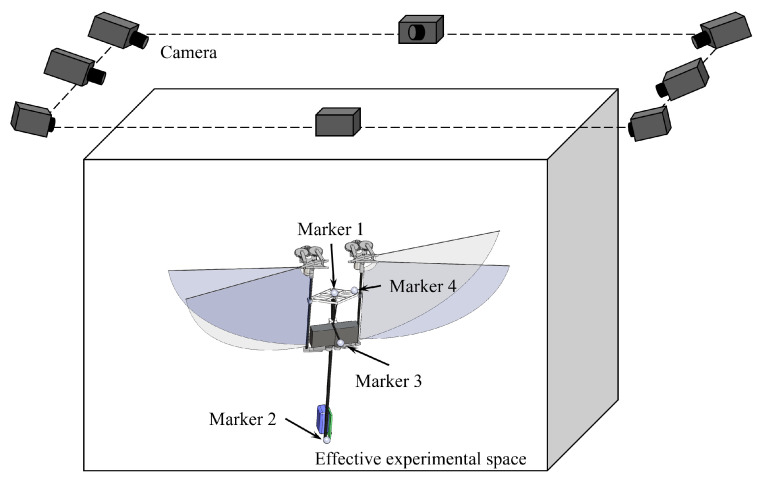
Vicon system setup for attitude angle (pitch θ, roll ϕ and yaw ψ), angular rates (*p*, *q*, *r*) and spatial position (x,y,z) real-time measurements using a frame rate of 150 Hz.

The Vicon motion capture system with eight cameras is used to conduct displacement tracking experiments on the controllable flight of the prototype. The experimental space is approximately 2 m length × 2 m width × 1.5 m height, and the shooting frame rate of a single camera is set to 150 fps, as shown in Figure 10. The prototype can take off quickly from the origin of the test space coordinates and reach a height of 1.5 m in 2 s at the fastest. It can achieve controllable hovering flight and control flapping wings to land in 3 s under remote control.

## 5. Results and Discussion

### 5.1. Aerodynamic Simulation

**Figure 11:**
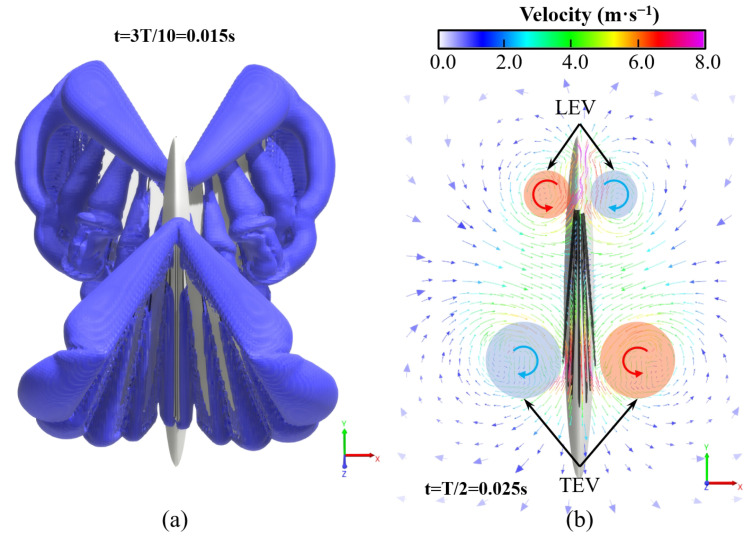
Simulation results of the wing flow field of the X-type FMAV: (**a**) at t = 0.015 s, the vorticity isosurface around the aircraft; (**b**) at t = 0.025 s, that is, when the wings clap together, vertical z-direction cutting plane, velocity vector field diagram at z = 0.1 m.

As shown in Figure 11a, during the flapping stroke of the aircraft, obvious edge vortices are generated around each segment of the wings, which can be divided into LEV and TEV, while WTV is generated at the wingtip. Due to the segmental flexibility, vortices will also appear in the gaps between each segment, which will not occur in the actual case of the entire wing. In this research, the scale of these vortices is small, and their impact on aerodynamic performance of the flight can be ignored. As shown in Figure 11b, the leading edge and trailing edge will produce a pair of significant vortex structures with reverse rotation during the flapping process of the X-type FMAV. We define them as LEV and TEV, which are important factors for the unsteady lift of the aircraft.

**Figure 12:**
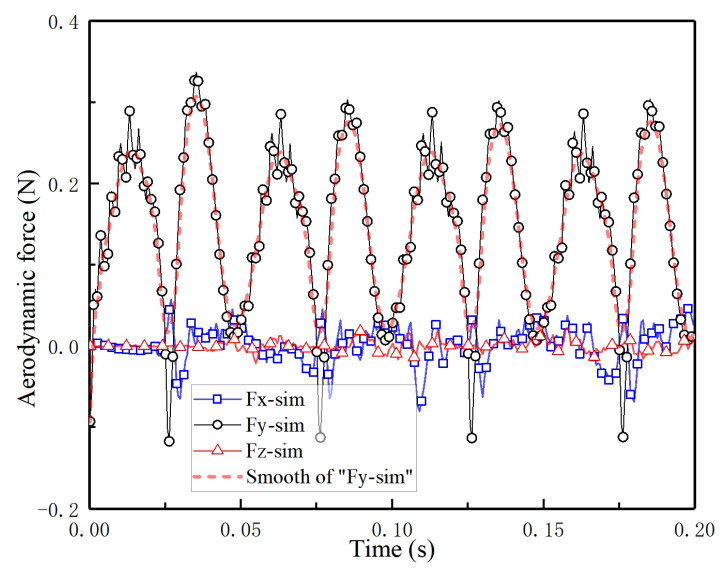
Instantaneous aerodynamic forces in X-, Y- and Z-axis for 4 cycles of hovering FMAV.

By CFD simulation, we obtain the instantaneous aerodynamic forces of the hovering FMAV in the X-, Y- and Z-axis. From the aerodynamic curve in Figure 12, the X-type FMAV can generate lift in each half cycle of wing clapping and flinging. Peak lift is generated around T/4 and 3T/4 respectively, and the maximum value of lift is 0.337 N near the middle position of the wing from closing to fully opening; the trough of lift is around 0 and T/2, and the minimum value of lift is −0.175 N, when the wings are fully clapped together.

The average lift generated by the X-type FMAV with rigid segmented model in one cycle can be calculated through CFD simulation to be 14.8 g at the determined flapping frequency. However, due to the flexibility of the actual wings, the designed wingspan, chord length, and airfoil deformation finally produce the aerodynamic force to meet the lift requirement of hovering. As shown in Figure 12, the instantaneous peak aerodynamic force after filtering is also not stable, up to 0.3 N.

**Figure 13:**
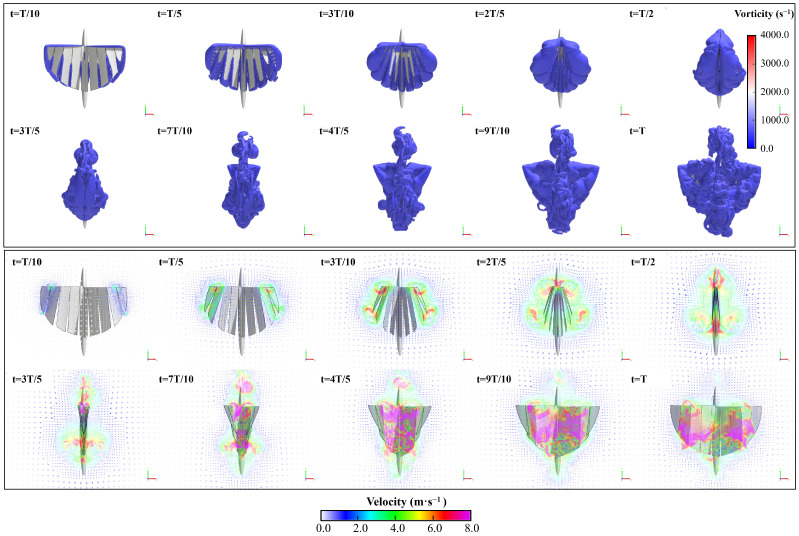
The cutting plane in the vertical z direction during the first flapping stroke of hovering flight, the frame-by-frame screenshot of vector velocity field at z = 0.1 m, and the flapping period is T = 0.05 s.

**Figure 14:**
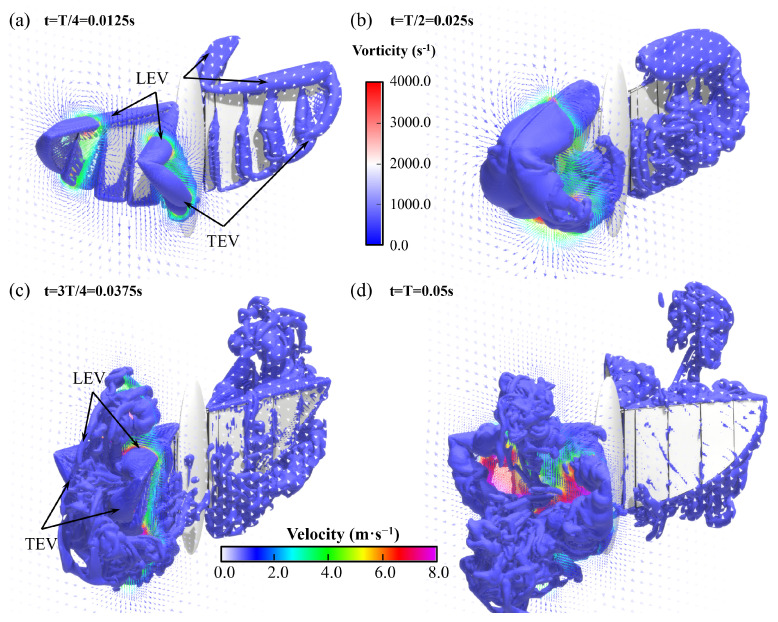
Visualization results of isosurface based on vorticity from an oblique downward 45° viewing angle at (**a**) t = T/4, (**b**) T/2, (**c**) 3T/4, and (**d**) T, respectively.

In the first flapping cycle, the flow field around the X-type FMAV is visualized using an equivalent surface with a vortex value of 700 s^−1^. As shown in Figure 13, the ipsilateral wing produces a starting vortex at the leading edge of the wing immediately after the start of flapping, and the vortex tube is visible. Then, a tapered leading-edge vortex gradually thickens from the wing root to the wing tip. Simultaneously, an obvious trailing edge vortex is generated at the trailing edge of the wing, and it reaches its maximum when the wings are completely flapped together. The trailing edge vortex was also very pronounced at the trailing edge of the wings and reached its maximum when the wings were fully flapped together. During the process from flapping together to opening to the maximum, the LEV and TEV were also generated on the inner side of the wing. Furthermore, the LEV and TEV generated in the first half stroke were captured separately to form a more chaotic vortex flow. As shown in Figure 14, the LEV and TEV are attached to one side of the wing during the whole stroke of flapping, which has an insignificant effect on the high lift generated by the vehicle. The influencing factors of the high lift mechanism of flapping-wing flight will be further explained via simulation and experiment.

**Figure 15:**
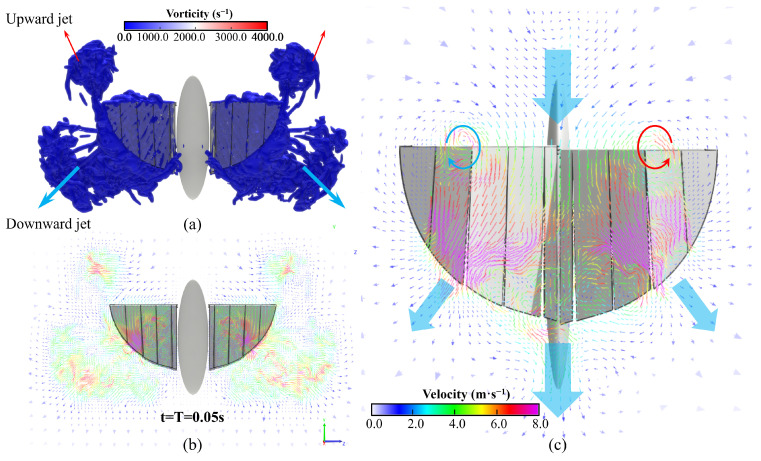
Simulation results of flow field at the end of the first cycle of hovering flight of the X-type FMAV: (**a**) the visualized image of front view vorticity isosurface; (**b**) the screenshot of velocity field at x = 0 m of cutting plane, perpendicul to the X-axis; (**c**) the screenshot of velocity field at z = 0.1 m of cutting plane, perpendicular to Z-axis.

For the X-type FMAV, the air is squeezed out by both wings during the flapping process. Through simulation, two types of jets flowing out in a flapping cycle were found, namely the inclined upward jet near the wing tip and the inclined downward jet near the trailing edge of the wing. The downward jet leads the upward one a lot in strength and velocity. As shown in Figure 15, the first period of the X-shaped flapping wing hovering flight ends: (a) the visualized image of front view vorticity isosurface; (b) the screenshot of the velocity field at x = 0 m of cutting plane, perpendicular to the X-axis; (c) the screenshot of the velocity field at z = 0.1 m of cutting plane, perpendicular to Z-axis.

### 5.2. Lift Generation and Manoeuvre Moment

**Figure 16:**
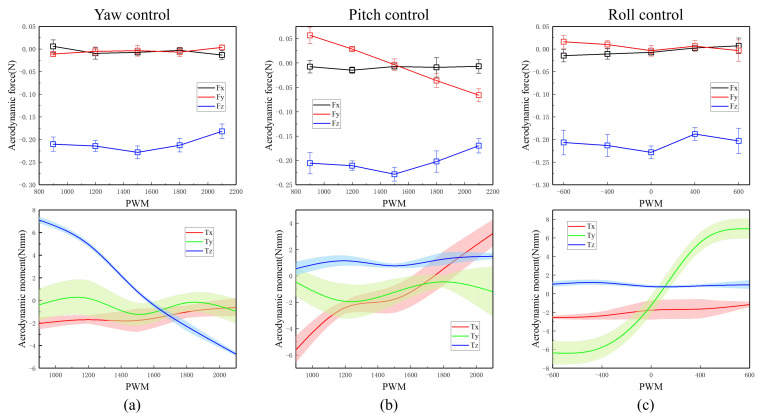
Generation of force and torques in three axes for a range of control inputs. (**a**) Yaw control; (**b**) Pitch control; (**c**) Roll control.

The experimental results between aerodynamic thrust and duty cycle of PWM control command is shown in Figure 16: (a) under yaw control, the aerodynamic force and torque generated by the prototype with continuous control duty cycle adjustment from 900 to 2100; (b) under pitch control, the aerodynamic force and torque generated by the prototype with continuous control duty cycle adjustment from 900 to 2100; (c) under roll control, the aerodynamic force and torque generated by the prototype with the duty cycle difference adjustment of the left and right motor drive control signals from −600 to 600.

A tailless model with independent wing control is adopted. While realizing torque control, the rudder surface of the tail wing is more sensitive to disturbances caused by wind gusts and reduces the stability of the system; tailless wing can avoid these shortcomings, but how to control the aircraft after leaving the traditional tail wing is also a problem. Thus, a vector control method based on the direct drive of the wings on both sides is proposed. Under the condition of reducing the mass and size, the sensitivity to gusts is reduced, and the stability is effectively improved. In the flapping wing mode, the wing-body movement is highly coupled. After the traditional tail and ailerons are canceled, the aircraft will find it difficult to control the attitude and position. The left and right independent drive wings improve the maneuverability of the aircraft, which not only makes the control modes more diverse, but also improves the robustness of the system.

The flexible segmented wing model is used for CFD simulation analysis. The flexibility of the chord direction of the wing can improve the aerodynamic characteristics of the wing under low Reynolds number, and increase the lift and thrust to a certain extent. In this research, the wings are made segmentally flexible in the chord direction, and each segment of the hinge is connected to the leading-edge drive rod. The corresponding law of motion is loaded for each section, and the effect similar to the flexibility of the film can be achieved. The airflow around the wing is displayed through a 3D visualization method. Combining the aerodynamic output results and the wing jet flow, the wing control parameters and geometric shape are optimized to provide theoretical support for subsequent prototype development.

**Figure 17:**
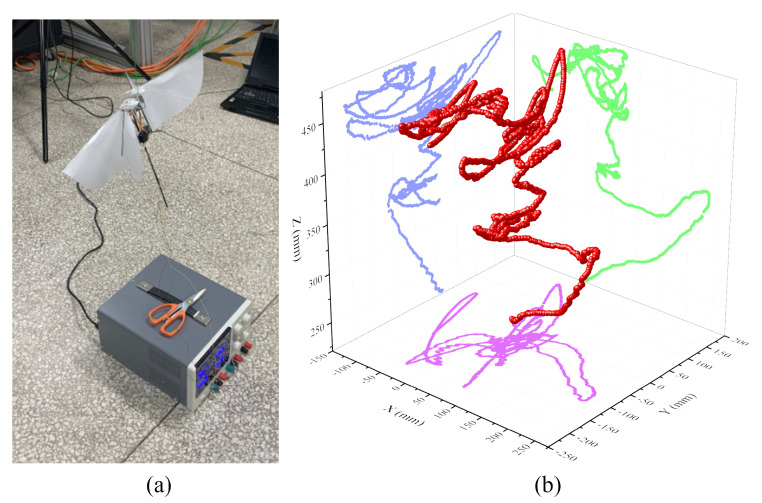
Free flight test of the prototype. (**a**) Take-off test of prototype with rope constraints; (**b**) A 3D trajectory plot of the X-type FMAV.

Figure 17 shows the movement tracking results of the prototype from take-off to hover. The prototype climbed from a takeoff position of 0.2 m to about 0.5 m in about 5 s. In the subsequent flight, the aircraft sensed the constantly changing Euler angle rate data in real time, and continuously corrected it through its own stability augmentation control system software and hardware to maintain the hovering state. This process lasted for about 6 s at a height of about 0.47 m. In the end, it can be verified that the remote control flight control of the prototype is feasible, and the stability and maneuverability of the whole flight still need to be further improved in the future.

For traditional fixed-wing and rotary-wing aircraft, the direction of lift is basically fixed for the fuselage axis. The flapping-wing air vehicle changes the magnitude and direction of lift and thrust in real-time by changing the angle of attack, flapping frequency, and amplitude of the wings. The biggest advantage of vector control is the direct force manipulation attitude control, which is different from the attitude control of the indirect aerodynamic torque of the tail rudder. It has a faster control response and is closer to the high maneuver attitude control of natural creatures. The parameters of the final flyable physical prototype are shown in Table 3.

## 6. Conclusions

A novel tailless X-type FMAV with left and right wings independently driven is proposed in this research. The prototype can produce pitch moments between −6 Nmm∼3 Nmm, yaw moments between −5 Nmm∼7 Nmm, and roll moments between −6 Nmm∼6 Nmm, while the two wings are flapping at frequencies around 20 Hz and producing a lift of at least 200 mN.

To reduce the size of the FMAV as much as possible and improve its agility, we designed and fabricated this four-wing tailless prototype. Its wing roots are connected with carbon fiber rods, and the wings on both sides are under independent vector control. While improving the stability of low-speed flight, it has a relatively high control bandwidth, enabling it to complete flight missions in complex environments. Based on LBM, the CFD simulation of a segmented and flexible wing model was carried out to verify the correctness and feasibility of the design. The flow field around the four wings is visualized based on the vorticity isosurface. There is obvious oblique downward jet flow in both the upstroke and downstroke. In addition, when the upper and lower wings clap together, a small upward jet appears near the wing tips. Dynamic experimental results provide a theoretical basis for the subsequent addition of functions and performance improvement.

Due to the high maneuverability and size advantages of this type of aircraft, the tailless X-type FMAV will be widely used in field surveys and detection of narrow and complex spaces. In particular, after various disasters such as earthquakes, landslides, and high-rise building fires, this novel vehicle can be directly used for accurate life detection and rescue in destroyed buildings and jungles.

In the future, we will consider increasing the flapping-wing amplitude to enhance aerodynamic thrust and improve the stability of the FMAV. At the same time, the wingspan of the prototype will be further reduced so that the prototype can implement multiple flight modes and be more agile, enabling it to complete operations in various small and complex environments.

## Figures and Tables

**Table 1:** Mass distribution of all components.

Components	Mass (g)	Percentage (%)
Coreless motors	3.88	14.4
Servos	6.24	23.2
Flapping-wing mechanism	4.08	15.1
Wings	0.84	3.1
Airframe	4.06	15.07
Onboard electronic system	2.66	9.9
Battery	5.02	18.6
Wiring, etc.	1.00	3.7
Total	26.94	100

**Table 2:** Parameters setup of CFD simulation.

Term	Value
Domain size	1.5m×2m×1m
Lateral boundaries	Periodic
Position	(0,−0.5,0)
Boundary conditions	Velocity
Reference density of air	1.225 kg · m^3^
Operating temperature	288.15 K
Dynamic viscosity of air	1.7894 × 10^−5^ Pa · s

**Table 3:** Parameters of the prototype.

Parameter	Value
Wing span	341 ± 0.1 mm
Wing chord	93.7 ± 0.1 mm
Mass	26.5 ± 0.1 g
Fuselage length	242 ± 0.1 mm
Flapping-wing amplitude	81° ± 2°
Flapping-wing frequency	20 Hz
Supply voltage	7.4 V
Gear reduction ratio	25:1

## Data Availability

Data are contained within the article.

## Abbreviations

The following abbreviations are used in this manuscript:

CFD	Computational Fluid Dynamics
LBM	Lattice Boltzmann Method
FMAV	Flapping-wing Micro Air Vehicle
FAV	Flapping-wing Air Vehicle
PIV	Particle Image Velocimetry
MEMS	Micro Electro Mechanical Systems
PWM	Pulse-Width Modulation
CoG	Center of Gravity
IMU	Inertial Measurement Unit
LEV	Leading-edge Vortex
WTV	Wing Tip Vortex
TEV	Trailing Edge Vortex
3D	Three-dimensional
FWSP	Flapping-wing Symmetry Plane
ESC	Electronic Speed Controller
